# Classification of Early and Late Mild Cognitive Impairment Using Functional Brain Network of Resting-State fMRI

**DOI:** 10.3389/fpsyt.2019.00572

**Published:** 2019-08-27

**Authors:** Tingting Zhang, Zanzan Zhao, Chao Zhang, Junjun Zhang, Zhenlan Jin, Ling Li

**Affiliations:** MOE Key Lab for Neuroinformation, High-Field Magnetic Resonance Brain Imaging Key Laboratory of Sichuan Province, Center for Psychiatry and Psychology, School of Life Science and Technology, University of Electronic Science and Technology of China, Chengdu, China

**Keywords:** resting-state fMRI, mild cognitive impairment, feature section, functional network, classification

## Abstract

Using the Pearson correlation coefficient to constructing functional brain network has been evidenced to be an effective means to diagnose different stages of mild cognitive impairment (MCI) disease. In this study, we investigated the efficacy of a classification framework to distinguish early mild cognitive impairment (EMCI) from late mild cognitive impairment (LMCI) by using the effective features derived from functional brain network of three frequency bands (full-band: 0.01–0.08 Hz; slow-4: 0.027–0.08 Hz; slow-5: 0.01–0.027 Hz) at Rest. Graphic theory was performed to calculate and analyze the relationship between changes in network connectivity. Subsequently, three different algorithms [minimal redundancy maximal relevance (mRMR), sparse linear regression feature selection algorithm based on stationary selection (SS-LR), and Fisher Score (FS)] were applied to select the features of network attributes, respectively. Finally, we used the support vector machine (SVM) with nested cross validation to classify the samples into two categories to obtain unbiased results. Our results showed that the global efficiency, the local efficiency, and the average clustering coefficient were significantly higher in the slow-5 band for the LMCI–EMCI comparison, while the characteristic path length was significantly longer under most threshold values. The classification results showed that the features selected by the mRMR algorithm have higher classification performance than those selected by the SS-LR and FS algorithms. The classification results obtained by using mRMR algorithm in slow-5 band are the best, with 83.87% accuracy (ACC), 86.21% sensitivity (SEN), 81.21% specificity (SPE), and the area under receiver operating characteristic curve (AUC) of 0.905. The present results suggest that the method we proposed could effectively help diagnose MCI disease in clinic and predict its conversion to Alzheimer’s disease at an early stage.

## Introduction

Alzheimer’s disease (AD) is a progressive neurodegenerative disorder that is clinically characterized by dementia and cognitive decline (1). According to the World Alzheimer’s Disease Report in recent years (2, 3), about 35.6 million people suffered from dementia in 2010, and global dementia care costs more than 600 billion US dollars or approximately 1% of the global GDP. Mild cognitive impairment (MCI), commonly characterized by slight cognitive deficits but largely intact activities of daily living (4, 5), is a transitional stage between the healthy aging and dementia that can be divided into EMCI and LMCI, according to extent of episodic memory impairment (6). Research has shown that individuals with MCI tend to progress to AD at a rate of approximately 10–15% per year (7). Jessen et al. (8) showed that the risk of LMCI conversion to AD is higher than that of EMCI. Identifying potentially high-sensitivity diagnostic markers that change with disease progression may assist the physician in making a diagnosis. If it is found at an early stage of MCI, patients can reduced the number of AD incidence by nearly one-third through rehabilitation exercises and medication (9). Unfortunately, sensitive markers vary with disease progression (6), and there are currently no definitive diagnostic biomarkers and effective treatments for AD (10). Thus, early detection of EMCI individuals increasingly attaches clinical importance to potentially delaying or preventing the transition from EMCI to LMCI. Many experts study the early diagnosis of AD diseases from the aspects of neuropsychology, chemistry, and medical imaging. In clinical practice, doctors use the neuropsychological scale to diagnose and treat patients because of their simple operation, less time, and doubts. MCI patients have a certain sensitivity when they are initially tested and are widely used by clinicians (11), but they are subjectively influenced by individuals. Individual differences are relatively large, and other diagnostic methods need to be combined to give the final diagnosis. In biochemistry, the levels of Aβ and p-tau proteins in CSF are important biomarkers (12). Studies have shown that the content of amyloid has increased before clinical symptoms appear, can be used for early prediction of clinical AD disease, but is not sensitive (13). The content of p-tau protein in AD patients is significantly increased, with high sensitivity and specificity, and has certain reference value in clinical diagnosis (14), but the detection of this index is traumatic, patients have certain rejection psychology, and clinical operation is more difficult. Compared with these methods, Hinrichs et al. (15) reported that clinical and imaging data [MRI and fludeoxyglucose (FDG-PET)] can be successfully combined to predict AD using machine-learning techniques. They found that the imaging modalities had a better performance in prediction of AD compared to clinical data.

Neuroimaging research shows that MCI and AD patients have significant disruption compared with healthy control group in either the structural network or functional network (16–19). Several studies using the electroencephalogram (EEG) (20) and MRI (16, 17) have found abnormal clustering coefficients and characteristic path lengths in the brain networks of AD patients, implicating a loss of small-worldness attributes and disrupted whole brain organization network. Liu and Zhang (21) also used functional networks to detect betweenness centrality alteration in MCI and compared with AD group, showed decreased in the amygdala and rolandic operculum, and increased in the frontal gyrus, parietal gyrus, and medial temporal lobe. However, for MCI patients, changes in the brain are very subtle (19, 20); therefore, few studies have examined the characteristics of whole brain networks in different stages of MCI patients. Xiang and colleagues (22) used functional brain networks to study the abnormal brain connection in MCI and reported that the clustering coefficient in EMCI is higher than that of LMCI, while the average shortest path in LMCI is longer than that of EMCI. Although the difference was not significant, this method of analyzing functional brain network differences might provide an effective feature reference for the classification to distinguish EMCI from LMCI.

Recently, several studies have demonstrated that the features obtained from functional brain network measures and machine learning approach based on rs-fMRI contribute useful information for more accurate classification. Chen et al. (23) used large-scale network (LSN) analysis with an AUC of 95% to classify subjects with amnestic mild cognitive impairment (aMCI n = 15) and cognitively normal (CN n = 20) subjects. Challis et al. (24) proposed GP-LR models and employed SVM with 75% accuracy to distinguish healthy subjects from subjects with amnesic mild cognitive impairment. Khazaee and colleagues (25) used time series to construct brain function network, and linear SVM classifiers were used to classify AD and normal people, which obtained 100% classification accuracy. This could be due to the small sample size, and the single variable Fisher Score feature selection algorithm was used. In another study, they extracted both temporal variabilities and spatial variabilities from dynamic connectivity networks (DCNs) as features, and integrate them for classification by using manifold regularized multi-task feature learning and multi-kernel learning techniques. The method they proposed yields the accuracy of 78.8% for LMCI and EMCI classification (26). It has been shown that combination of the graph theory with machine learning approach on the basis of rs-fMRI can accurately classify patients with MCI, patients with AD, and normal subjects (22, 23).

However, most of the studies pooled EMCI and LMCI groups into a single larger MCI group (24, 25, 27), and few studies investigated utility of rs-fMRI to distinguish two groups (25). In addition, Zuo et al. (28) divided the BOLD signal into five bands: full-band (0.01–0.08 Hz), slow-2 (0.0198–0.25 Hz), slow-3 (0.073–0.0198 Hz), slow-4 (0.027–0.073 Hz), and slow-5 (0.01–0.027 Hz). Brain activity of MCI patients has significant differences in the posterior cingulate, hippocampus, and medial prefrontal regions in the slow-4 band and slow-5 band, and the classification of MCI by frequency division achieved a better classification result (29, 30). Thus, the combination of functional brain networks and frequency division provides a new direction for classifying MCI patients.

In the current study, we aim to evaluate the efficacy of a classification framework to distinguish EMCI from LMCI by using the effective features derived from functional brain network of three frequency bands during Rest States. On the basis of classification result to find high-sensitivity features, we can better understand why sensitive markers in brain region vary with disease progression. We supposed that providing appropriate treatment and cognitive training for patients’ high-sensitivity brain region at different stages of the disease might be preventing the progression of AD transformation.

Firstly, we preprocessed the signal and divided it into three frequency bands (full-band: 0.01–0.08 Hz; slow-4: 0.027–0.08 Hz; slow-5: 0.01–0.027 Hz) at Rest. Then, we constructed functional brain network by calculating Pearson’s correlation coefficients between time series of all pairs of the brain regions and thresholded it to an undirected binary network. Several graph-theoretic parameters (global efficiency, local efficiency, characteristic path length, clustering coefficient, and small-worldness) were selected to measure the characteristics of functional brain networks. Nodal characteristics were examined at a high discriminative range of sparsity from 8 to 20%. At the feature selection step, we employed three different algorithms for selecting optimal feature. To obtain unbiased results, support vector machine (SVM) classifiers with nested cross validation were used for classification. Finally, we compared the performances of three feature selection methods from classification results. We supposed that classification results may be influenced by different bands and the classification results may be the best in the slow-5 band.

## Materials and Methods

### Participants

Data used in the preparation of this article were obtained from the Alzheimer’s Disease Neuroimaging Initiative (ADNI) database (adni.loni.usc.edu). The ADNI was launched in 2003 as a public-private partnership, led by Principal Investigator Michael W. Weiner, MD. The primary goal of ADNI has been to test whether serial magnetic resonance imaging (MRI), positron emission tomography (PET), other biological markers, and clinical and neuropsychological assessment can be combined to measure the progression of mild cognitive impairment (MCI) and early Alzheimer’s disease (AD).

The demographic data of the datasets are listed in **Table 1**. This study included 33 early MCI (EMCI) patients (average age 71.69 years, 19 female) and 29 late MCI (LMCI) patients (average age 70.73 years, 13 female). In the ADNI project, MCI diagnostic criteria included 1) Mini-Mental State Examination (MMSE) scores between 24 and 30, 2) a memory complaint, objective memory loss measured by education adjusted scores on the Wechsler Memory Scale Logical Memory II, 3) a Clinical Dementia Rating (CDR) of 0.5, and 4) absence of significant levels of impairment in other cognitive domains, essentially preserved activities of daily living, and an absence of dementia. As shown in ADNI project, the MCI stage was divided into EMCI and LMCI. Detailed diagnostic criteria of EMCI and LMCI: Both are characterized by evidence of AD biomarker abnormalities, with EMCI patients showing milder cognitive deficits. In terms of neuropsychological criteria, EMCI is defined as a performance 1–1.5 SD below the mean in one episodic memory test, identifying intermediate level of subtle memory impairment between normal cognition and MCI (31). In **Table 1**, we listed the p values of a Chi-Square test of gender and a two-sample t-test of age, CDR, and MMSE. We can see that gender, age, and MMSE have no signification differences for EMCI vs. LMCI.

**Table 1:** Demographic data of EMCI vs. LMCI subjects.

Variable	EMCI n=33	LMCI n=29	p-value
Gender (M/F)	14/19	16/13	0.316
Age	71.69±5.74	70.73±5.90	0.519
CDR	0.5	0.5	1
MMSE	28.12±1.65	27.17±2.20	0.058

Values represent mean ± SD. MMSE, Mini-Mental State Examination; CDR, Clinical Dementia Rating. Chi-Square test was used for gender comparison. Two-sample t-test was used for age, CDR, and MMSE comparison. P > 0.05 indicates two groups have no significant differences.

### Data Acquisition

All subjects underwent structural and functional MRI scanning on 3T Philips scanner according to the ADNI acquisition protocol (32). The structural images were acquired with T1-weighted magnetization prepared rapid acquisition gradient echo (MPRAGE) sequences (170 slices; TR = 3,000 ms; TE = 30 ms; matrix = 256 × 256; voxel size = 1.2 × 1.0 × 1.0 mm^3^; flip angle = 9°). rs-fMRI scans were acquired with a T2*-weighted echo planar imaging (EPI) sequence with the following scanning parameters: 48 slices; TR = 3,000 ms; TE= 30 ms; matrix = 64 × 64; voxel size = 3.313 × 3.313 × 3.313 mm^3^; flip angle = 80°.

### Preprocessing

rs-fMRI data preprocessing was performed using software MATLAB 2013a (MathWorks, Inc, https://www.mathworks.com) and Data Processing Assistant for Resting-State Functional MR Imaging (DPARSF) (33) toolbox and Statistical Parametric Mapping software (SPM8) (34) package (http://www.fil.ion.ucl.ac.uk/spm) and Resting-State fMRI Data Analysis Toolkit (35) (REST; http://restfmri.net) for each subject. The preprocessing steps were as follows:

(1) For signal stabilization and to allow the participants to adapt to the environment, the first 10 EPI volumes of the fMRI images were discarded.
(2) Slice-timing correction for interleaved acquisition.
(3) Realignment for head movement compensation by using a six-parameter rigid-body spatial transformation. None of the subjects were excluded on the basis of the criterion with head motion limited to less than 2 mm or 2°.
(4) Each of structural MRI images was coregistered to the mean functional image by using a linear transformation, and the transformed structural images were segmented into grey matter (GM), white matter (WM), and cerebrospinal fluid (CSF) by using a unified segmentation algorithm. The functional images were normalization to Montreal Neurologic Institute (MNI) space.
(5) Spatial smoothed with 6 mm FWHM Gaussian kernel and linear detrending were implemented as well.
(6) The global mean signal, six head motion parameters, CSF, and WM signals were also removed as nuisance covariates to reduce the effects of motion and non-neuronal blood oxygenation level-dependent (BOLD) fluctuations (36, 37).
(7) Low frequency signals were divided into full-band (0.01–0.08 Hz), slow-4 (0.027–0.08 Hz), and slow-5 (0.01–0.027 Hz).

### Functional Network Construction

The nodes of the brain network were defined by parcellation of the whole brain into 90 distinct regions using the automated anatomical labeling (AAL) atlas, which is a gross functional subdivision of the cortex (38). The time series of voxels within each of the 90 ROIs was averaged, and the resulting signal was used as the node. The edges were constructed by calculating Pearson’s correlation coefficients between time series of all pairs of the brain regions. We applied Fisher’s r-to-z transform on raw undirected connectivity matrix of the three bands to improve the normality of the partial correlation coefficients (18, 39). By definition, this matrix is symmetric with a zero diagonal (no self-connections) (40). To determine the available edges, each individual’s brain network sparsity is thresholded as a binary matrix, where the edges are 1 if the weights of the two ROIs are larger than a given threshold, and 0 otherwise. The threshold represents the network connection cost, defined as the ratio of the suprathreshold connections relative to the total possible number of connections in the network (41). There is no straightforward rule for the definition of the single sparseness threshold, and different sparsenesses lead to different experimental results (17, 37). In this study, each network was examined for the range of costs from 8% to 20%, at 1% intervals. We performed a search over different thresholds to find the optimal threshold value (42). In order to generate effective network characteristics, statistically significant differences in network parameters between the two groups of patients under different sparsity levels were calculated.

### Graph Theory Parameters

All graph theory parameters were computed and analyzed using Matlab 2013a (MathWorks, Inc) scripts and matlab_bgl (https://github.com/dgleich/matlab-bgl)

The undirected connectivity matrix in three bands for each subject was used to calculate different graph metrics. To obtain efficient features and avoid feature largely redundancy, we first computed five global graph measures on the undirected graphs. The global graph measures are as follows: global efficiency, local efficiency, characteristic path length, clustering coefficient, and small-worldness (43). We performed two sample *T* test on five graph metrics of two groups subjects. In **Supplementary Figures 1**,**** **2**, and **3**, results showed that global efficiency, local efficiency, clustering coefficient, and characteristic path length had significant differences in slow-5 band. Although there are no obvious differences in slow-4 and full-band, the trend is similar to slow-5 band.

### Feature Extraction

**Figure 1:**
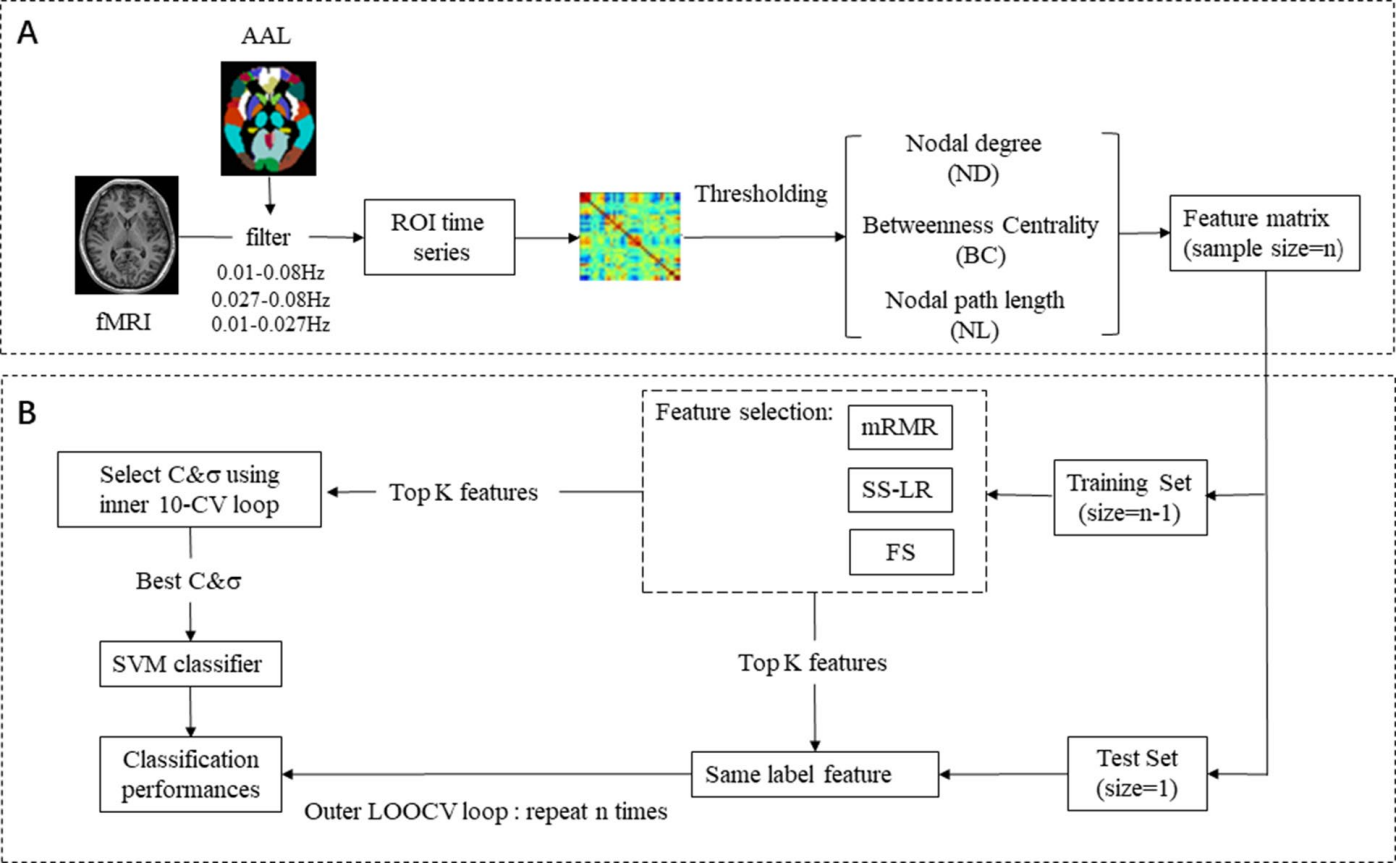
EMCI and LMCI classification framework. **(A)** Raw data preprocessing, feature extraction, and feature selection process. **(B)** Classification: SVM classifier with nested cross validation is implemented for classification.

In feature extraction section (**Figure 1A**), 270 nodal features [nodal path length (NL), nodal degree (ND), and betweenness centrality (BC)] were employed for subsequent analysis. For ND, BC, and NL, we utilize 270 features in each band, a total of 270 × 3 = 810 features. In brief, for a given node *i*, NL, ND, and BC were defined as follows:

(1) $$L_{i}=\frac{\sum_{j\neq i\in V}L_{ij}}{\left(V-1\right)}$$

(2) $$\;K_{i}=\sum_{j\in V}b_{ij}$$

(3) $$B_{i}=\sum_{i\neq j\neq m\in V}\frac{S_{jm}(i)}{S_{jm}}\;$$

where *L* *_ij_* represents the minimum number of edges between node *i* and *j*, *V* is the size of a graph, *b* *_ij_* is the connection status between the node *i* and *j*, *S* *_jm_*, represents the number of shortest path lengths between node *m* and *j*, and *S* *_jm_*(*i*) represents the number of shortest paths through the node *i* between node *m* and *j*. Intuitively, path length *L* *_i_* measures the speed of the message that passes through a given node, and the degree of an individual node *Ki* is equal to the number of links connected to that node, and the greater the *B* *_i_* is, the more important the node *i* is to the information communication in the network, thus reflecting the level of interaction in the network.

### Feature Selection

As shown in **Figure 1A**, we selected 270 features from three types of network features (NL, ND, and BC) for three frequency bands (slow-4, slow-5, full-band) of each subject, respectively. In particular, we took integrate feature sets from three bands into a new feature named all band for subsequent analysis. There is no doubt that feature selection is a wonderful choice that degrades redundancy in feature, reduces training-testing time, and improves classification performance. Here, three sorts algorithm were applied to feature selection.

#### Minimal Redundancy Maximal Relevance Feature Selection Algorithm (mRMR)

Here, we utilized mRMR for feature selection that was first proposed by Ding and Peng (44) in 2005. mRMR can commendably solve tradeoff problem between feature redundancy and relevance that uses mutual information as a feature correlation measure factor (45). Given two random variables *X* and *Y*, Mutual information between them is defined as:

(4) $$I(X,Y)=\iint p(x,y)\log\frac{p(x,y)}{p(x)p(y)}dxdy$$

where *p*(*x*) and *p*(*y*) refers to probabilistic density functions and *p*(x, y) is their joint probability density function.

Max-Relevance is to search features satisfying that is defined as:

(5) $$\max D(S,c),D=\frac{1}{\left|S\right|}\sum_{x_{i}\in S}I(x_{i};c)$$

*S* refers to feature set with m features {*x* *_i_*} and *c* is the class. The relevance of a feature set *S* for the class *c* is defined by the average value of all mutual information values between the individual feature *x* *_i_* and the class *c*

Min-Redundancy is defined as:

(6) $$\min R(S),R=\frac{1}{{\left|S\right|}^{2}}\sum_{x_{i},x_{j}\in S}I(x_{i},x_{j})$$

Formula is used to select mutually exclusive features. The criterion combining the above two constraints is called “minimal-redundancy-maximal-relevance” (mRMR). The mRMR is defined as:

(7) $$mRMR=\underset{S}{max}\left\{\frac{1}{\left|S\right|}\sum_{x_{i}\in S}I(x_{i};c)-\frac{1}{{\left|S\right|}^{2}}\sum_{x_{i},x_{j}\in S}I(x_{i},x_{j})\right\}$$

#### Sparse Linear Regression Feature Selection Algorithm Based on Stationary Selection (SS-LR)

Given a data set *T* = (*X*, *Y*), where X = (*x*_1_, *x*_2_, … , *x* *_n_*)*^T^* ∈ *R* *^n^* ^×^ *^m^* is the sample, *Y* = (*y*_1_, *y*_2_, …, *y* *_n_*)^T^ ∈ *R* *^n^* ^×^^1^ is its associated sample real label, *n* is the number of samples, and *m* is the number of features of each sample. The model of linear regression can be defined as:

(8) f(X)=Xw

where *w* = (*w*_1_, *w*_2_, …, *w* *_n_*) ∈ *R* *^m^* ^×^^1^ is the coefficient in the linear regression, and *f*(*X*) is the prediction label vector obtained by discriminating the unknown sample. Let *L*(*w*) be the loss function of linear regression, and then the function is as shown in Equation (9):

(9) $$L(w)=\underset{w}{\min}\frac{1}{n}||f(X)-Y|{|}_{2}^{2}$$

In order to control the complexity of the model, an *L*_1_ regularization term is usually added after the loss function, and the expression after regularization is added:

(10) $$L(w)=\underset{w}{\min}\frac{1}{n}||f(X)-Y|{|}_{2}^{2}+|\lambda |w|{|}_{1}$$

where $||w|{|}_{1}=\sum_{i=1}^{m}w_{i}$, λ >0 is a regularization parameter in control of the model. As λ increases, the sparseness of the function becomes larger, that is, in front of some feature attributes. The coefficient becomes 0, that is, linear regression with *L*_1_ regularization can be used for feature selection. In this paper, the SLEP package (46) was used to solve sparse linear regression. To solve the problem of proper regularization, we employed subsampling or bootstrapping to apply the stability selection for robust feature selection (47). In this study, the range is 0.05 < λ < 0.3, and the step size is 0.005.

#### Fisher Score

Fisher Score is a univariate feature selection algorithm. The feature with the identification criteria should satisfy the variance of the features in the selected sample of the same category as small as possible. On the contrary, the variance between the features in the different categories of samples should be as large as possible. It is helpful for high classification accuracy of subsequent prediction results. Suppose *m* *_i_* represents the average of the* i*-th feature in all samples, *m*_1_ *_i_* represents the average of the *i*-th feature in the one sample, and *m*_2_ *_i_* represents the average of the *i-*th feature in another sample. The Fisher Score value for each feature in a two class problem is defined as (48):

(11) $$FS(i)=\frac{n_{1}{\left(m_{1i}-m_{i}\right)}^{2}+n_{2}{\left(m_{2i}-m_{i}\right)}^{2}}{\left(n_{1}\sigma_{1i}^{2}+n_{2}\sigma_{2i}^{2}\right)}$$

In formula, *n*_1_ is the number of samples in the first type of sample, *n*_2_ is the number of samples in the second type of sample, and $\sigma_{1i}^{2}$ is expressed as the i-th feature in the first type of sample. The variance in $\sigma_{2i}^{2}$ is expressed as the variance of the *i*-th feature in the second type of sample.

#### SVM Classifier

After the feature selection stage, the support vector machine (SVM) algorithm was applied to classification that is supervised machine learning algorithm using the LIBSVM toolbox (49), with radial basis function (RBF) and an optimal value for the penalized coefficient C (a constant determining the tradeoff between training error and model flatness). The RBF kernel was defined as follows:

(12) $$K(X_{1},X_{2})=exp\left(-\frac{\left||X_{1}-X_{2}|\right|}{2\sigma^{2}}\right)$$

where *x*_1_ and *x*_2_ are two eigenvectors, and σ is the width parameter of the REF kernel. The classification framework flow chart is shown in **Figure 1B**. We used nested cross-validation (CV) to obtain unbiased estimates and select the optimal SVM model. On the training set, the optimal hyperparameters (C and σ) by a grid-search and a 10-fold CV (inner loop) was employed. For the outer loop, the leave-one-out cross validation (LOOCV) was used and repeated N times (N = 62). We selected one sample as the validation set and the remaining samples as feature selection and classifier training set for each fold of the outer CV. This operation was repeated until all subjects used once as test sample. Finally, we used the held-out sample to evaluate the performance of the training classifier. Area Under Curve (AUC) is defined as the area enclosed by the coordinate axis under the ROC curve. The larger the AUC score, the more likely the current classification algorithm is to rank the positive samples in front of the negative samples, which is a better classification. Most researchers have now adopted AUC for evaluating the predictive capability of classifiers since AUC is a better performance metric compared to accuracy (50).

To evaluate the performance of the classification results, these established measures were defined as follows:

(13) $$\begin{array}{l} Sensitivity=\frac{TP}{TP+FN},\\ Accurary=\frac{TP+TN}{TP+TN+FP+FN},\;\\ Specificity=\frac{TN}{TN+FP} \end{array}$$

where TP, TN, FP, and FN represent true positive, true negative, false positive, and false negative, respectively. According to traditional rules, we considered a correctly predicted EMCI as a true positive and LMCI as a true negative (51).

## Results

### Classification Results

**Figure 2:**
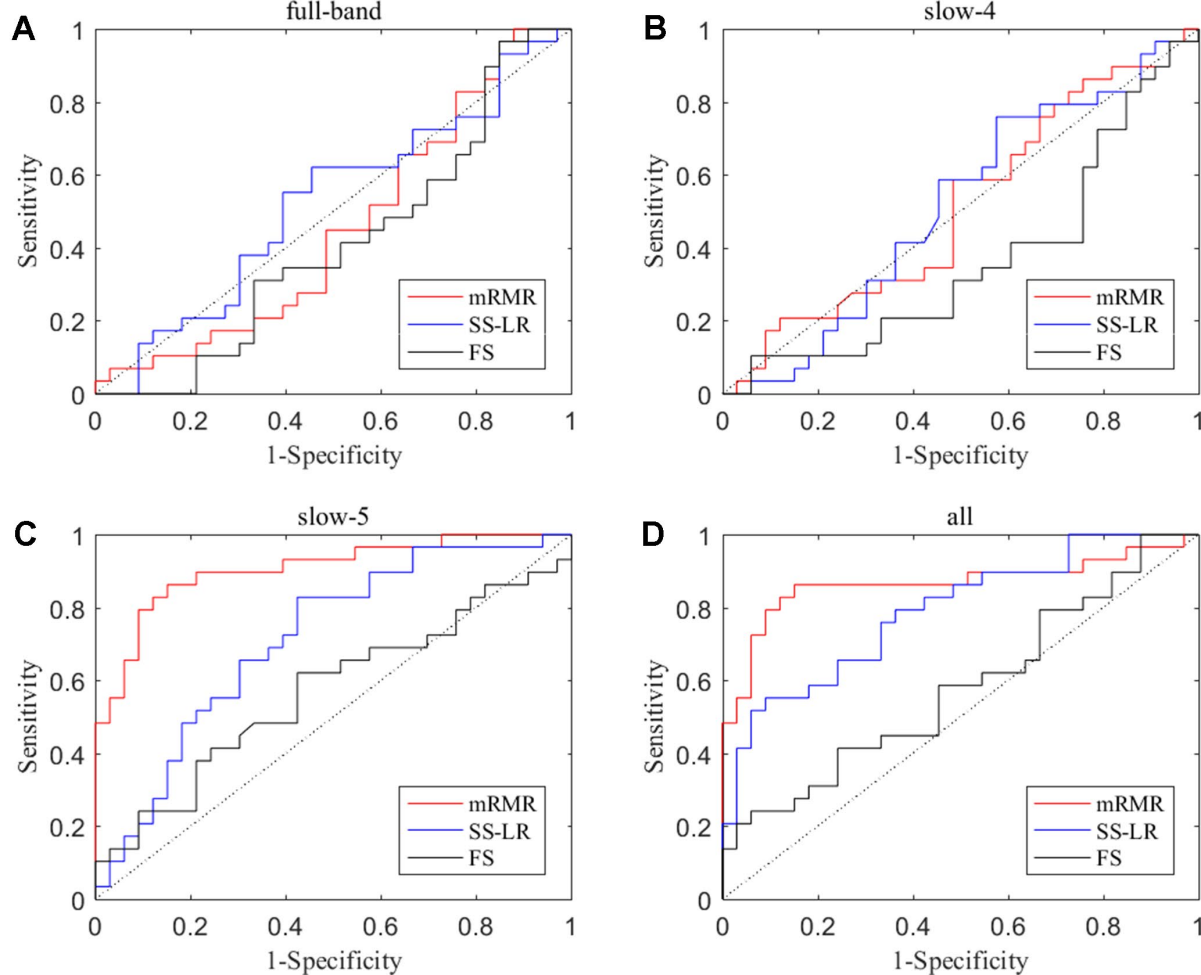
ROC curves for the three algorithms using the top 10 nodal features **(A)** full-band, **(B)** slow-4 band, **(C)** slow-5 band, and **(D)** all band.

In the absence of a specific threshold value, the features of the four frequency bands (slow-4, slow-5, full-band, and all band) are selected by the mRMR, SS-LR, and FS in the Cost = 8–20%. Through a series of classification results with threshold, the AUC scores in the slow-5 band is significantly higher than that in the other frequency bands. By comparison, we found that the classification results in the slow-5 band are the best and stable under threshold value of Cost = 15%. The following results are analyzed and discussed in the threshold of Cost = 15%. The receiver operating characteristic (ROC) curves and classification results are depicted in **Figure 2** and **Table 2**.

**Table 2:** Classification results performance of different methods using the top 10 features.

Frequency band	mRMR				SS-LR				FS			
	ACC (%)	SEN (%)	SPE (%)	AUC	ACC (%)	SEN (%)	SPE (%)	AUC	ACC (%)	SEN (%)	SPE (%)	AUC
Full-band	40.32	27.59	51.52	0.454	53.23	44.83	60.61	0.523	48.39	34.48	60.61	0.411
Slow-4	51.61	24.14	75.76	0.512	50.00	41.38	57.58	0.512	37.10	34.48	39.39	0.363
Slow-5	83.87	86.21	81.82	0.905	64.52	58.62	69.70	0.713	58.06	44.83	69.70	0.569
All	82.26	72.41	90.91	0.865	67.74	65.52	69.75	0.789	54.84	43.86	63.64	0.579

ACC, accuracy; SEN, sensitivity; SPE, specificity.

For the mRMR algorithm model, the all band achieved a classification accuracy of 82.26% (sensitivity = 72.41%, specificity = 90.91%, AUC = 0.865). The slow-5 resulted in a higher accuracy of 83.82% (sensitivity = 86.21%, specificity = 81.82%, AUC = 0.905). Specifically, we obtained slightly lower levels of accuracies for full-band and slow-4 (40.32% and 51.61%, respectively) compared to the classification of all-band vs. slow-5. For the SS-LR algorithm model, the all-band achieved a higher accuracy of 67.74% (sensitivity = 65.52%, specificity = 69.75%, AUC = 0.789). The slow-5 resulted in accuracy of 64.52% (sensitivity = 58.62%, specificity = 69.70%, AUC = 0.713). For the FS algorithm model, the all-band achieved a classification accuracy of 54.84% (sensitivity = 43.86%, specificity = 63.64%, AUC = 0.579). The slow-5 resulted in a higher accuracy of 58.06% (sensitivity = 44.83%, specificity = 69.70%, AUC = 0.569).

**Figure 3:**
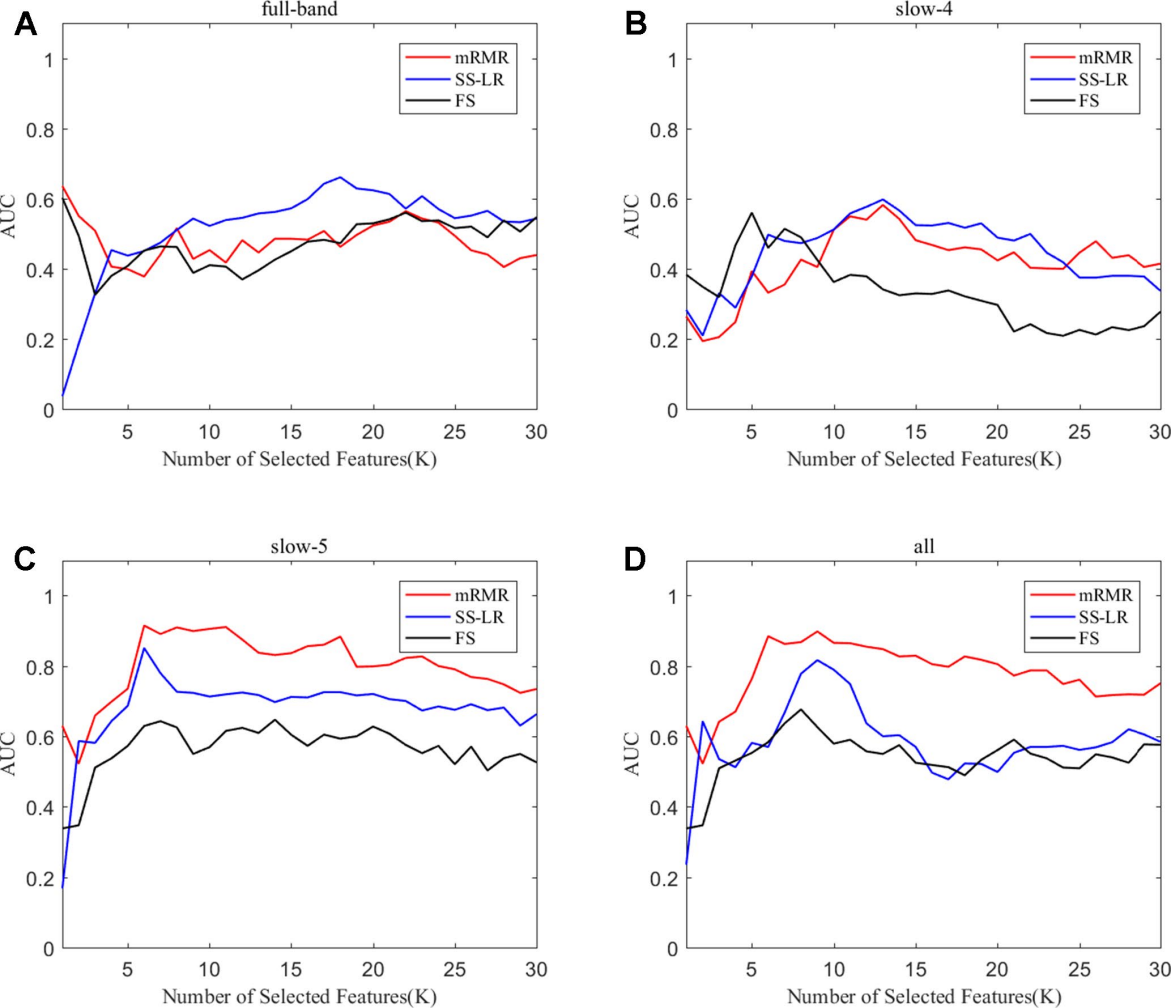
Subgraphs **(A)**, **(B)**, **(C)**, and **(D)** represent AUC curves with the number of features K of full-band, slow-4, slow-5, and all band.

To prove the effect of the number of selected features, we used the top K features (K = 1, 2, ..., 30) for classification. The classification performances and AUC scores are shown in **Figure 3**, respectively. The AUC curves appeared stable after the top 8 features, and the best classification results are depicted in the slow-5 band and all band. The AUC scores of slow-5 band and all band are higher than those in the full-band and slow-4 band. For the slow-5 band, the AUC scores increased as the number of selected features increased, and the AUC curve of the mRMR algorithm is highest, followed by SS-LR, and the lowest is FS. In all band, the highest among AUC curves is mRMR, and SS-LR and FS are comparable. The AUC curves for the three algorithms in the slow-4 and full band are relatively low and relatively messy, which cannot be distinguished by observation. In summary, it can be seen from the classification results of three feature selection algorithms that suitable algorithm may improve the classification effect.

#### Comparing Classification Results Based on Different Feature Selection Methods

In order to compare whether the classification effects of the classifiers under the different feature selection algorithms are significantly different, the McNemar test is used to compare the classification results of two different feature selection algorithms respectively. All statistics were computed with Matlab2013a platform.

**Figure 4:**
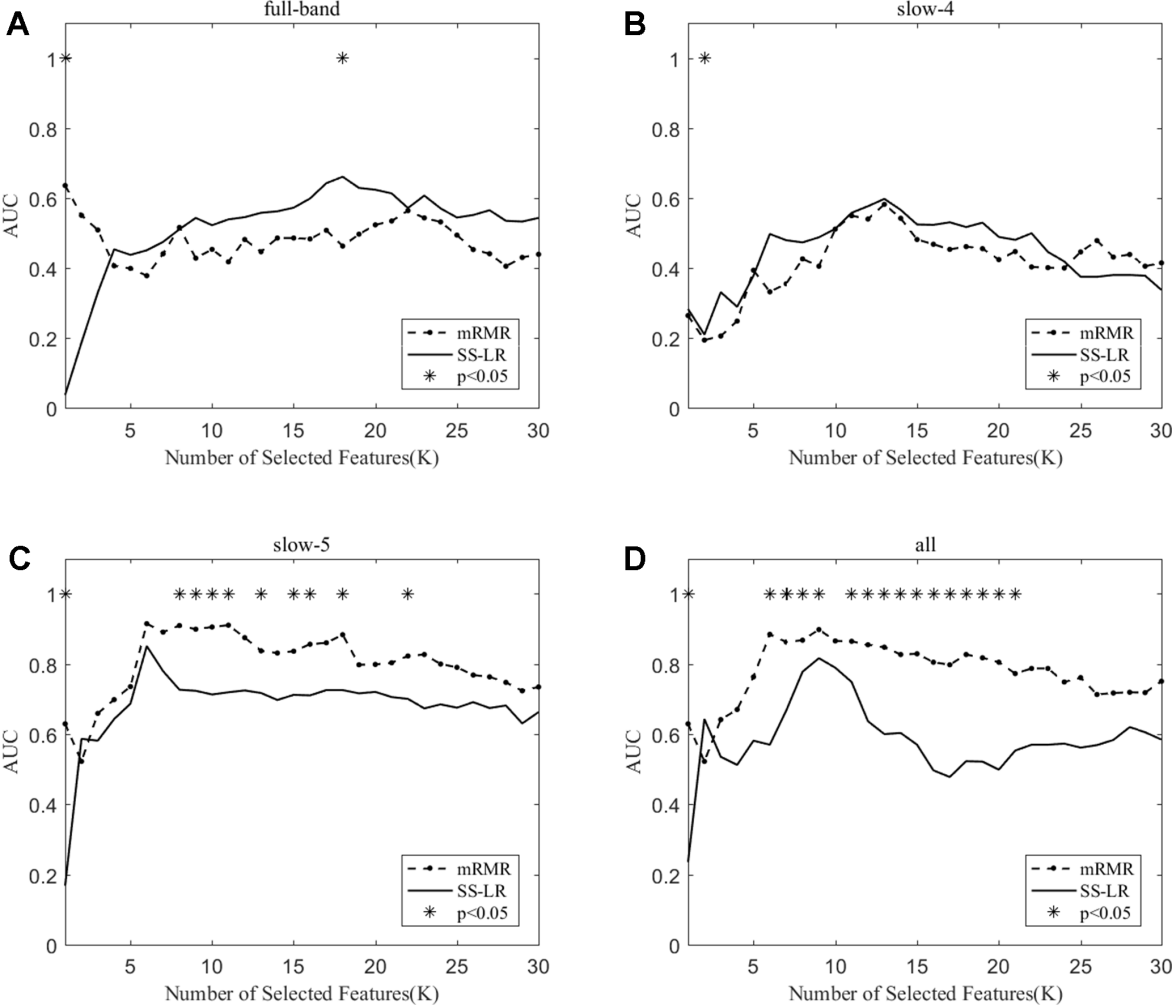
The AUC with the number of features under the mRMR and SS-LR algorithms; * indicates a significant difference in the classification results under the two algorithms.

When the number of features is K=10, the classification results and the p value obtained by using the mRMR and SS-LR algorithms are shown in **Tables 2** and **3**, and **Figure 4** shows the AUC scores with the number of features under the mRMR and SS-LR algorithm. As shown in **Table 3**, we compared the results of the four frequency bands using the mRMR and SS-LR feature selection algorithms, and only the classification results of the slow-5 band showed significant differences (p = 0.006). The AUC scores of mRMR were significantly higher than SS-LR (**Table 2**). Using the mRMR algorithm, the slow-5 band achieved the best AUC scores (AUC = 0.905), while the all band performed slightly lower (AUC = 0.865), and full-band and slow-4 band classification results both performed poor. Using the SS-LR algorithm, the classification result shows that the all band obtained the best results (AUC = 0.789), while the slow-5 band performed slightly lower (AUC = 0.713), with poor performance in full-band and slow-4 band. From **Figure 4**, the classification results of the two algorithms in full-band and slow-4 band showed almost no significant differences with the K value increase and both of the AUC scores are relatively low, and can hardly be classified correctly. The classification result obtained by using the mRMR algorithm in the slow-5 band is obviously better than that of the SS-LR algorithm. In the range of 6<K<11, the mRMR curve tends to be ﬂat, while as the K value increases, the RMR curve shows a gentle decline. The SS-LR curve tends to be flat over the entire K value range. The classification result of the mRMR algorithm in all band is significantly better than the SS-LR algorithm, and the curve of the mRMR algorithm is flatter than the curve of the SS-LR algorithm.

**Table 3:** Comparison of classification results between different feature selection methods.

Frequency band	Sig. (mRMR VS SS-LR)	Sig. (mRMR VS FS)	Sig. (SS-LR VS FS)
Full-band	0.1356	0.3827	0.6056
Slow-4	1.000	0.1508	0.0990
Slow-5	0.006	0.0014	0.4795
All	0.0665	0.00048	0.0990

The “Sig.” column gives the p-value. Results with Sig. value < 0.05 are treated as nominally significant.

**Figure 5:**
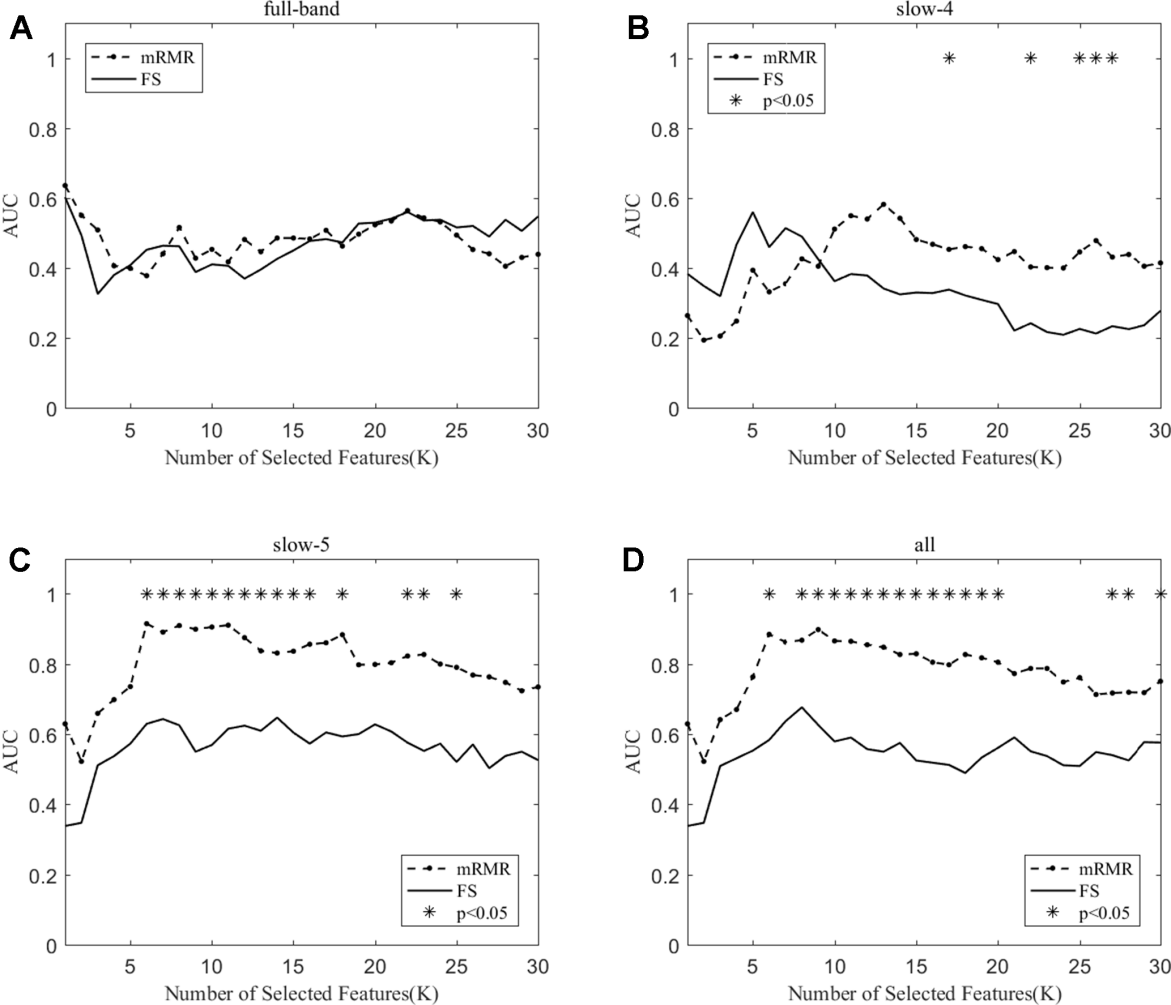
The AUC with the number of features under the mRMR and FS algorithms; * indicates a significant difference in the classification results under the two algorithms.

We compare the classification performance of the mRMR algorithm and the FS algorithm, and the results are shown in **Table 3** and **Figure 5**. For the slow-5 band and all band in **Table 3** **,** the classification results obtained by the mRMR algorithm and the FS algorithm showed significant differences, and the difference in all band is relatively large (p = 0.00048). We found no significant difference between the two algorithms in the full-band and slow-4 band. Using the mRMR algorithm, the AUC scores of the slow-5 band were higher, the all band were second, and the full band and slow-4 band were the worst. In four frequency bands, the classification results obtained by the mRMR algorithm were better than that of FS. As can be seen from **Figure 5**, in the full-band, the AUC scores obtained by the two algorithms have no significant difference within the all range, and there were significant differences in the slow-4 band within the several range (K = 17,22,25,26,27). In the slow-5, the AUC scores obtained by the mRMR curve was significantly larger than the AUC scores of the FS curve, and the mRMR curve shows a downward trend with the K value increase, while the FS curve tends to be stable. In the all band, the AUC scores obtained by the mRMR curve were significantly larger than FS, and both curves show a gentle downward trend.

**Figure 6:**
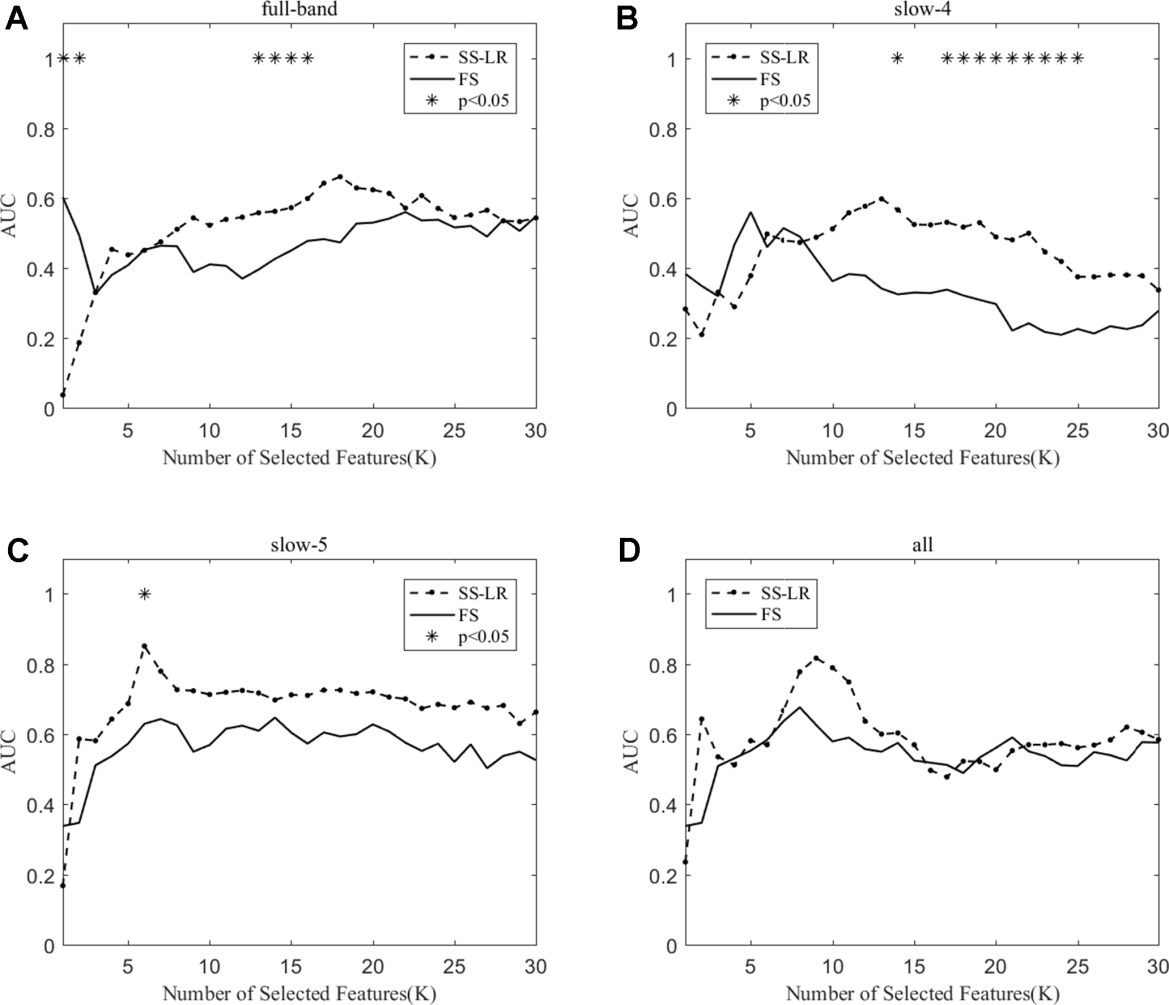
The AUC with the number of features under the SS-LR and FS algorithms; * indicates a significant difference in the classification results under the two algorithms.

As shown in **Table 3**, the classification performance obtained by the two algorithms has no significant difference in each frequency band, but the classification results obtained by the SS-LR algorithm was higher than the FS algorithm. As can be seen from **Figure 6**, there were significant differences in AUC scores in the full-band (K = 1,2,13,14,15,16), slow-4 (K = 14,17,18, ..., 25), and slow-5 (K = 6) band, and there was no significant difference in all band. Among the four frequency bands, the AUC scores are relatively higher in the slow-5 band than that in the other three bands. In the slow-5 band, the trend of the two curves was relatively ﬂat, and the waveforms of the two curves vary in other frequency bands. It can be seen from the classification results of different frequency bands that dividing the frequency band may improve the classification effect (52–56).

In brief, the classification results obtained by using the mRMR algorithm in the slow-5 band was the best, followed by the classification result obtained by using the mRMR algorithm in all band, while the classification results obtained by using the two algorithms in the full-band and slow-4 band are relatively poor. Hence, the next work is only for discussion and analysis of slow-5 and all band.

### Highly Sensitive Characteristic

This section lists the top 10 features in slow-5 band and all band obtained by the mRMR algorithm. Details on the specific characteristics of the selected features, the location and number of the AAL brain regions, and the number of selected times can be found in **Tables 4** and **5**. The features selected using the mRMR algorithm contain all the attributes, where the nodal path length (NL) attribute contains five features, and the betweenness centrality (BC) attribute contains three features, and nodal degree (ND) attribute contains two features. We found that the nodal path length attribute contributed 50% to identifying different stages of MCI.

**Table 4:** Distribution of features selected using the mRMR algorithm in the slow-5 band.

Networks attribution	Number	Region (AAL)	Selected times	Frequency (%)
ND	85	Left Middle temporal gyrus	62	100
BC	90	Right Inferior temporal gyrus	62	100
BC	83	Left Superior temporal gyrus	62	100
ND	72	Right Caudate nucleus	62	100
NL	79	Left Heschl gyrus	62	100
NL	53	Left Inferior occipital gyrus	62	100
NL	17	Left Rolandic operculum	62	100
BC	45	Left cuneus	61	>80
NL	22	Right Olfactory cortex	54	>80
NL	1	Left Precentral gyrus	53	>80

**Table 5:** Selected feature distributions in the integrated all band using the mRMR algorithm.

Networks attribution	Frequency band	Number	Region (AAL)	Selected times
NL	Full-band	53	Inferior occipital gyrus	62
ND	Slow-5	85	Left Middle temporal gyrus	62
BC	Slow-5	90	Right Inferior temporal gyrus	62
BC	Slow-5	83	Left Superior temporal gyrus	62
ND	Slow-5	72	Right Caudate nucleus	62
NL	Slow-5	53	Left Inferior occipital gyrus	62
NL	Slow-5	79	Left Heschl gyrus	61
NL	Slow-5	17	Left Rolandic operculum	61
BC	Slow-5	45	Left cuneus	60
NL	Slow-5	22	Right Olfactory cortex	23

**Figure 7:**
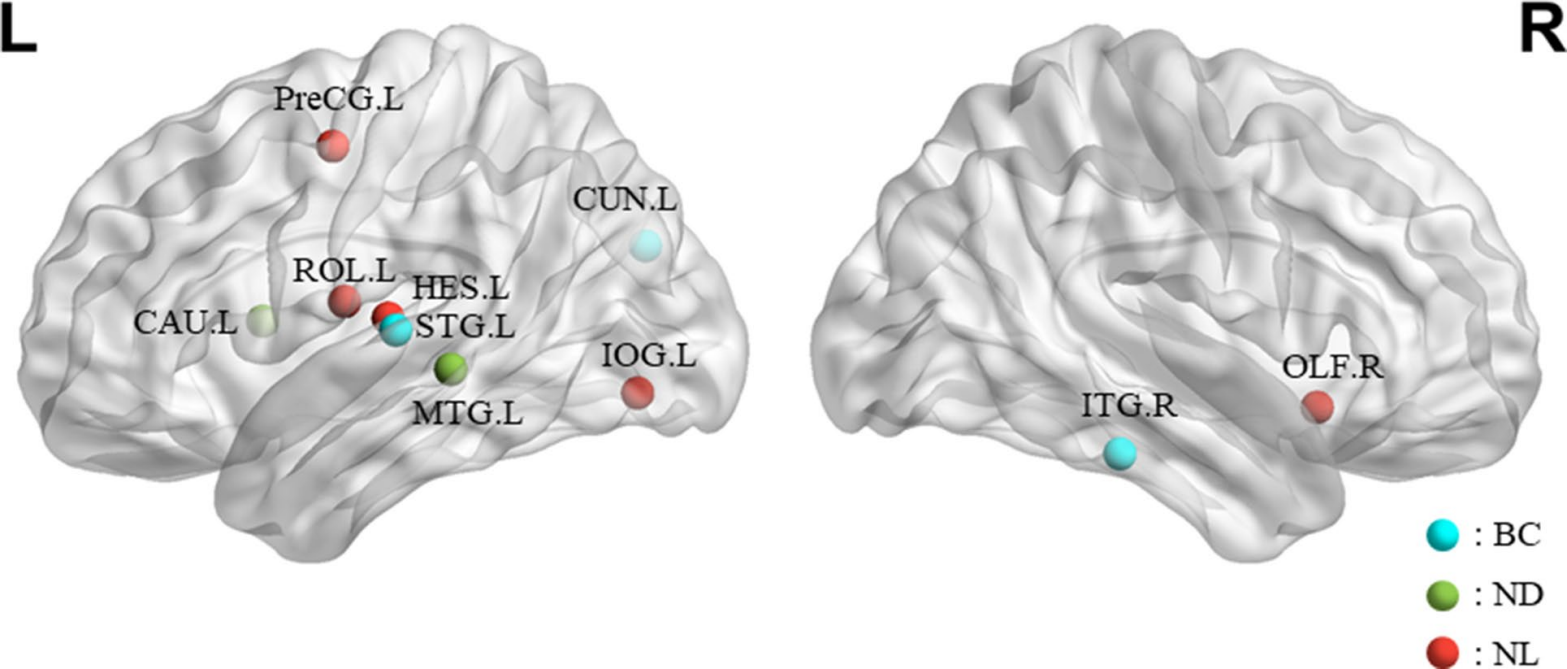
The location and networks attribution of top 10 brain regions, listed in **Table 4**, which might be affected in early stage of MCI in sagittal views. The blue ball represents BC, the red ball represents NL, and the green ball represents ND.

The features selected (listed in **Table 4** and **Figure 7**) show roughly similar features to two frequency bands and include the left middle temporal gyrus (l-MTG), the right inferior temporal gyrus (r-ITG), the left superior temporal gyrus (l-STG), and the right caudate nucleus (r-CAU), left heschl gyrus (l-HES), left inferior occipital gyrus (l-IOG), left rolandic operculum (l-ROL), left cuneus (l-CUN), right olfactory cortex (r-OLF), and the left precentral gyrus (lPreCG). These seven brain regions were 100% selected 62 times, and three brain regions were located in the temporal lobe region. The remaining three brain regions were also selected at a frequency of more than 80%.

In addition, we also list the features selected by the mRMR algorithm in the all band. The features of all band are combined by the full-band, slow-4, and slow-5 band. As can be seen from **Table 5**, except for one nodal path length attribute feature comes from the full-band band, other features are from the slow-5 band, and these features from the slow-5 band are consistent with the features selected separately from the slow-5 band. The features of the slow-4 band are not selected, and most of the features are selected from the slow-5 band, indicating that the information in the slow-5 band that distinguishes between EMCI and LMCI is highly sensitive characteristic.

## Discussion

In this paper, we employed the method of constructing brain function network to classify EMCI and LMCI in the case of sub-band. Although the all band contains all features of the three frequency band, the best classification effect was achieved in the slow-5 band (ACC=83.87%, AUC=0.905) by using the feature selection method of mRMR (**Table 2**). It can be seen that the analysis in brain function network properties of the two groups in **Supplementary Figures 1**, **2**, and **3**, there are significant differences in the network attributes of the two groups in the slow-5 band, so that both of highly sensitive features and best classification results in the slow-5 band can be inferred. These results suggest that low frequency obtained by division frequency might achieve a better classification result. In addition, compared with the SS-LR and FS feature selection algorithms, the features selected by the mRMR algorithm have higher classification performance and the classification effect is more stable with the number of features increases. It suggests that selecting the appropriate feature selection method for the data set can help improve the classification accuracy. From the demographic data of the two groups (**Table 1**), there is no significant difference between MMSE and CDR, which indicates that the neuropsychological scale could not distinguish the patients with EMCI and LMCI in the clinic. Our classification framework demonstrates that efficient feature extraction and selection can effectively improve the classification of EMCI and LMCI.

As shown in **Supplementary Figures 1**, **2**, and **3**, we used graph theory to calculate and analyze brain network functional differences between EMCI and LMCI. The results show that there are no significant differences in functional network properties between EMCI and LMCI in the slow-4 band. In the full-band, the global efficiency of LMCI is significantly higher than EMCI in a small part of the threshold, while the characteristic path length of LMCI is significantly longer than that of the small part of the threshold. In the slow-5 band, the global efficiency, the local efficiency, and the average clustering coefficient of LMCI are significantly higher than those of EMCI, respectively. Similarly, the LMCI characteristic path length is significantly longer than EMCI under most threshold values. Consistent with our findings, it has been shown that LMCI converters and EMCI converters showed a decreased path length and mean clustering compared with the MCI stables. Specifically, EMCI converters showed a decreased clustering coefficient, transitivity, modularity, and small-worldness compared with the LMCI converters in the Cost = 5–17% threshold range (57). These findings align with Zhou’s report (16) that MCI converters experience the worst local efficiency during the converting period to AD; however, the stables have highest local and global efficiency. They suggested that the abnormal brain network indicates a compensatory mechanism of local and global efficiency in these MCI stables.

As listed in **Tables 2** and **3**, the classification results show that the features selected by the mRMR algorithm have higher classification performance than those selected by the SS-LR and FS algorithms. For the mRMR algorithm, the classification results obtained in slow-5 band is more stable than that of slow-4 and full-band. As shown in **Table 6**, the results of constructing brain function network classification EMCI and LMCI in slow-5 band is better than that of other studies constructing brain network (26, 52, 58–62). Meanwhile, most previous methods (63–66) obtained accuracy <70% that constructed brain networks only considered structural feature. In brief, this study provides a valuable insight into the prediction of EMCI and LMCI conversion, and revealed that graph measures of resting-state fMRI are a potential predictor for classification. Our results suggested that brain activity in the slow-5 band carries more disease information and the top 10 selected features have high sensitivity for more efficient classification, compared with the slow-4 band and the full band. High sensitivity of functional network features, the frequently band segmentation of the signal, and the choice of the feature selection algorithm are critical to the classification.

**Table 6:** Classification performance of different methods to distinguish different stages of MCI.

Article	Method	Cohort	ACC (%)	SEN (%)	SPE (%)	AUC
**This paper**	Proposed	EMCI/LMCI (33/29)	83.87	86.21	81.21	0.905
**Biao Jie (** 26 **)**	Spatio-temporal interaction patterns of dynamic connectivity networks	EMCI/LMCI (56/43)	78.8	74.4	82.1	0.783
**Seyed Hani Hojjatia (** 52 **)**	Graph theory and machine learning approach (mRMR, FS)	MCI-C/MCI-NC(18/62)	91.4	83.24	90.1	N/A
**Mohammed Goryawala (** 58 **)**	fMRI volumes and neuropsychological scores	EMCI/LMCI (114/91)	73.6	74.3	72.7	N/A
**Heung-Il Suk (** 59 **)**	93 features from a MR image and the same dimensional features from a FDG-PET image.	MCI-C/MCI-NC (43/56)	74.04	58	82.67	0.696
**Zhang and Shen (** 60 **)**	MRI, PET and cognitive scores, Leave-one-out cross-validation	MCI-C/MCI-NC (38/50)	78.4	79.0	78.0	0.768
**Moradi et al. (** 61 **)**	MRI, age and cognitive measures 10-fold cross-validation	sMCI/pMCI (100/164)	81.72	86.65	73.64	0.902
**Ardekani et al. (** 62 **)**	Hippocampal volumetric integrity (HVI) from structural MRI scans RF with 5,000 trees	sMCI/pMCI (78/86)	82.3	86.0	78.2	N/A

The best multivariate predictors of MCI conversion are shown for each study. ACC, accuracy; SEN, sensitivity; SPE, specificity; AUC, area under the curve; FDG-PET, fluorodeoxyglucose positron emission tomography; RF, Random forest.

Previous studies demonstrated connection abnormalities in the temporal lobe region in patients with AD (15, 17). Liu et al. (67) also reported decreased complexities in lPreCG, STG, and MTG in familial AD. In agreement with these studies, we found that the temporal lobe region may be affected during the early stage of MCI. Specifically, we found that the betweenness centrality in the right inferior temporal gyrus (r-ITG) and the left superior temporal gyrus (l-STG) and the nodal degree in the left middle temporal gyrus were discriminative for separating EMCI from LMCI (**Tables 4** and **5**). The MTG has the highest selectivity in the feature selection section. These results are consistent with other reports that MTG is the most important brain regions in the AD lesion (68, 69). The MTG is located in the default network in the resting state network. Studies (70, 71) have shown that the default network in the resting state network of AD patients is abnormal compared to the normal elderly. Other studies have shown that a large amount of Aβ deposition is found in the temporal lobe region, indicating that this brain region is an important region in the development of AD disease (14). All of these results suggest that changes in the structure and function of the MTG region are more sensitive to the development of AD disease. Some of other sensitive brain regions, such as l-CUN and r-ITG, were also reported in previous study using PE method to analyze the complexity of the same ADNI dataset (72). Studying the structural and functional network results of AD suggests that cognitive impairment in patients may be caused by abnormal connections between different brain regions in the temporal lobe (70, 73). The area of the ITG plays an important role in maintaining language fluency (74). Hojjati and colleagues (52) demonstrated capability of rs-fMRI to predict conversion from MCI to AD by identifying affected brain regions (i.e., l-CUN, l-ROL, l-STG, r-CAU, r-ITG) underlying this conversion, and they proposed the ITG is an essential area in the verbal fluency circuit. Therefore, they suggested that these results might be indicative of disruption in communication between the ITG and other regions involved in this cognitive function in early stage of AD. For the caudate nucleus (CUN) region, Persson’s study found that larger caudate nucleus volume in AD patients and further discussed this region possibly serving as a mechanism for temporary compensation (75). Consistent with this structural MRI finding, our results revealed the functional connection abnormalities of r-CAU in early AD. Niu et al. (69) revealed significant differences in the OLF.R, l-IOG, l-MTG, and other brain regions on multiple time scales for four stages of AD. Khazaee and colleagues (19) suggested that patients with AD experience disturbance of l-ROL, r-ITG, and l-STG in their brain network as AD progresses. Our findings converge nicely with what has been suggested by the previous MRI studies (76–78), and these selected brain regions have been shown to be related with MCI conversion.

In summary, the highly sensitive characteristic found that the features selected using the mRMR algorithm in the integrated all band and slow-5 band are overlapping, indicating that the information contained in the slow-5 band is more distinguishable. Moreover, selected brain regions carry more disease information with highly sensitive characteristic leading to more efficient classification. The important role of temporal lobe in MCI disease has been widely recognized. We suggested that the other regions (Right caudate nucleus, Left Heschl gyrus, Left Inferior occipital gyrus, Left Rolandic operculum, etc.) deserve researchers pay attention to explore the role of these brain regions in the MCI disease.

## Conclusion

In this study, we investigated the efficacy of a classification framework to distinguish individuals with EMCI and LMCI by using the effective features derived from functional brain network of three frequency bands during Resting States. Without requiring other new biomarkers, our approach shows that the functional network features selected by mRMR algorithm improves the discrimination between EMCI and LMCI, compared with those selected by the SS-LR and FS algorithms. Moreover, the selected brain regions and frequency band are interpretable and consistent with previous studies. By comparing classification results, we found that the selected slow-5 band shows more stable and better performances compared with other bands. Ultimately, such a classification framework for the whole brain overall organization could substantially extend our understanding on the classification of MCI, shedding light on the novel potential diagnostic markers (highly sensitive features) located brain regions. This study has several limitations. A larger sample size and the consideration of including other degrees of severity in AD series and dementias in future work are essential to evaluate the variability and stability of functional networks for classification results. Another limitation related to network characteristics is the construction of undirected networks, ignoring the direction of information dissemination. Moreover, other findings indicated that any comparison of network parameters across studies must be made with reference to the spatial scale of the nodal parcellation (79); hence, we will evaluate the results of Power-264 brain regions for our method. The multimodality classification approach yields statistically significant improvement (at least 7.4%) in accuracy over using each modality independently (39). Further studies are needed to integrate information from structural and functional connectivity networks for improving classification performance.

## Data Availability

Publicly available datasets were analyzed in this study. This data can be found here: http://adni.loni.usc.edu/wp-content/uploads/how_to_apply.

## Author Contributions

LL helped in calculation and manuscript writing. TZ was in charge of the data analysis and manuscript writing. ZZ and CZ helped in speeding up the data analysis. JZ and ZJ corrected the manuscript. All authors reviewed the manuscript.

## Funding

This research was supported by grants from NSFC (61773092, 61673087, 61773096) and 111 project (B12027).

## Conflict of Interest Statement

The authors declare that the research was conducted in the absence of any commercial or financial relationships that could be construed as a potential conflict of interest.
