# Supplementary material for: Classification of Early and Late Mild Cognitive Impairment Using Functional Brain Network of Resting-State fMRI
Source: Front Psychiatry. 2019 Aug 27;10:572. doi: 10.3389/fpsyt.2019.00572 (PMC6727827; doi:10.3389/fpsyt.2019.00572)
Supplement: Supplementary file 1 [file DataSheet_1.pdf]

Supplementary Material

Brain Network Analysis Results

Tingting Zhang[1], Zanzan Zhao[1], Chao Zhang[1], Junjun Zhang[1], Zhenlan Jin[1], Ling Li[1*], for the Alzheimer's Disease Neuroimaging Initiative[†]

*** Correspondence:** Ling Li: liling@uestc.edu.cn

Here we analyzed the full range of costs from 8 to 20%, at 1% intervals. By calculating and analyzing the differences in the functional network attributes of the two groups of EMCI and LMCI in the slow-5 band, slow-4, full-band.

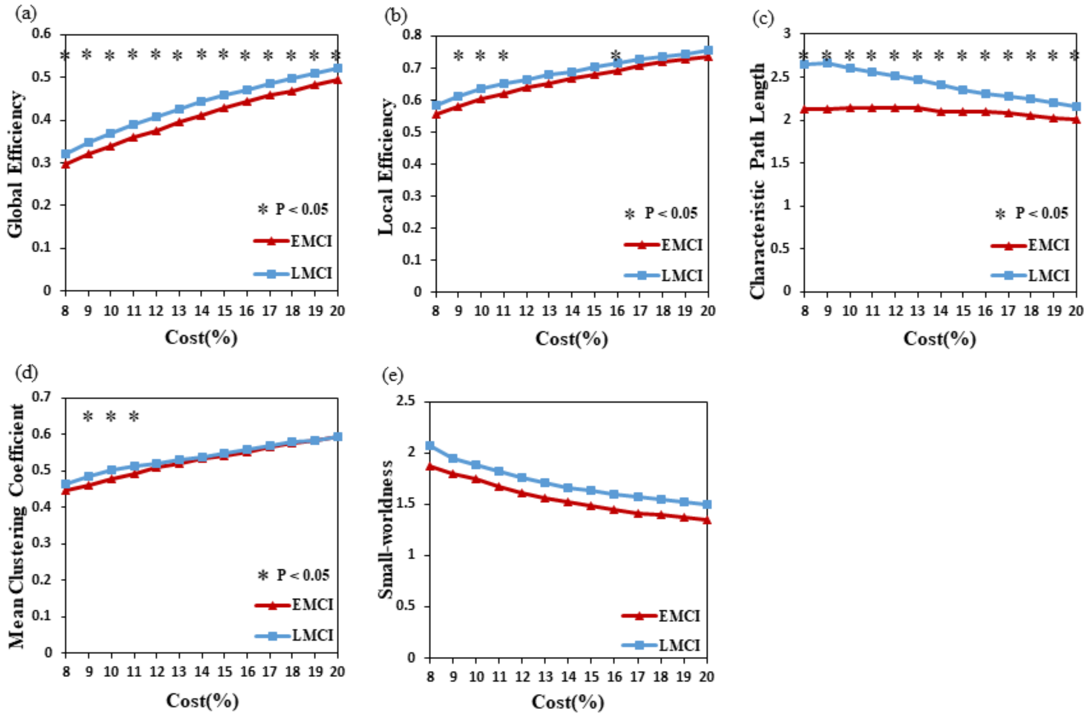

Supplementary Figure 1 | The comparison of network parameters of the slow-5 band between EMCI and LMCI. (a-e) The global efficiency, local efficiency, characteristic path length, mean clustering coefficient and small-worldness of the subjects for each sparsity (from 8 to 20% with a step size of 1%). The x-axis represents the degrees of sparseness, the y-axis represents different attribute values, the blue line represents LMCI, the red line represents EMCI, and * indicates the significant differences in two groups (2-sample t test, $P < .05$). The global efficiency, local efficiency, characteristic path length and mean clustering coefficient of patients with LMCI were significantly higher than those of EMCI across most costs. The small-worldness attributes did not show significant differences between EMCI and LMCI in the slow-5 band.

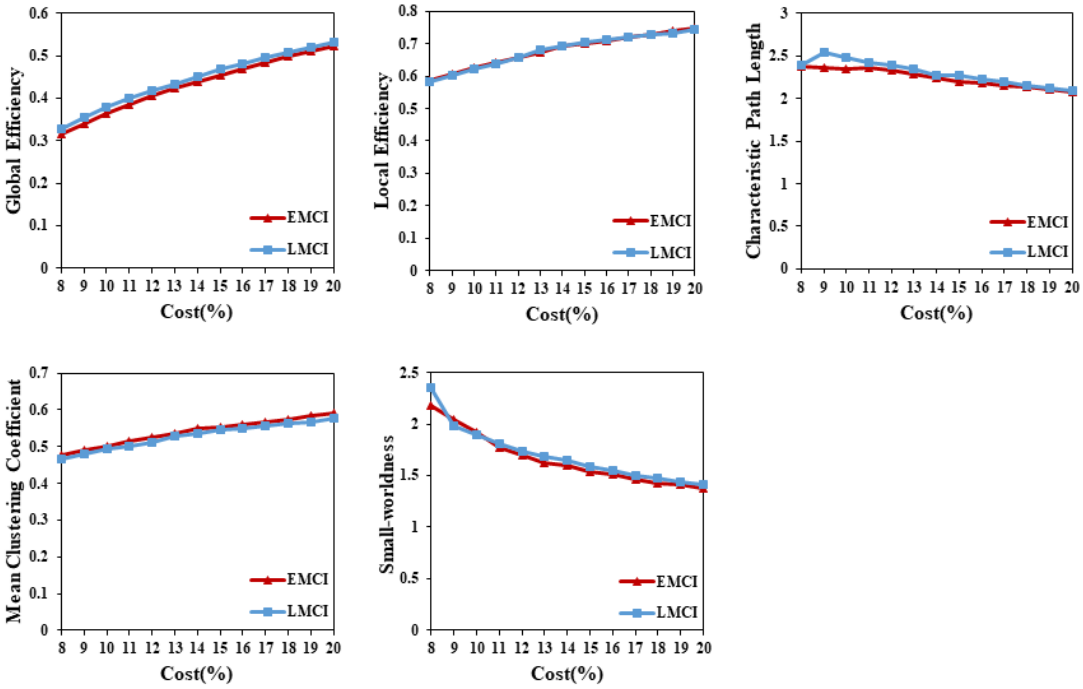

Supplementary Figure 2 | The comparison of network parameters of the slow-4 band between EMCI and LMCI. (a-e) The global efficiency, local efficiency, characteristic path length, mean clustering coefficient and small-worldness of the subjects for each sparsity (from 8 to 20% with a step size of 1%). The x-axis represents the degree of sparseness, the y-axis represents different attribute values, the blue line represents LMCI, the red line represents EMCI, and * indicates the significant differences in two groups (2-sample t test, P < .05). All network parameters did not show significant differences between EMCI and LMCI in the slow-4 band.

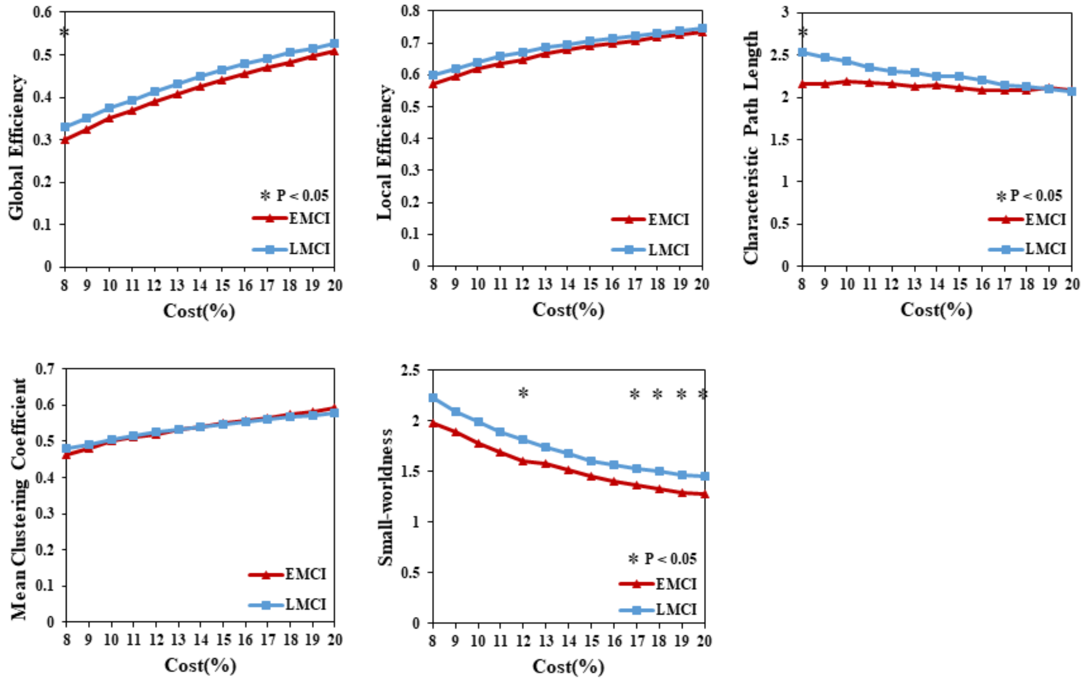

Supplementary Figure 3 | The comparison of network parameters of the full-band between EMCI and LMCI. (a-e) The global efficiency, local efficiency, characteristic path length, mean clustering coefficient and small-worldness of the subjects for each sparsity (from 8 to 20% with a step size of 1%). The x-axis represents the degree of sparseness, the y-axis represents different attribute values, the blue line represents LMCI, the red line represents EMCI, and * indicates the significant differences in two groups (2-sample t test, $P < .05$). The small-worldness attributes of patients with LMCI were significantly higher than those of EMCI across most costs. The global efficiency and characteristic path length of patients with LMCI were significantly higher than those of EMCI only at Cost = 8%. The local efficiency and mean clustering coefficient did not show significant differences between EMCI and LMCI in the full-band.
